# Cost‐effectiveness analysis of an active search to retrieve HCV patients lost to follow‐up (RELINK‐C strategy) and the impact of COVID‐19

**DOI:** 10.1111/jvh.13686

**Published:** 2022-05-23

**Authors:** Elena Vargas‐Accarino, Joan Martínez‐Campreciós, Raquel Domínguez‐Hernández, Ariadna Rando‐Segura, Mar Riveiro‐Barciela, Francisco Rodríguez‐Frías, Ana Barreira, Adriana Palom, Miguel Ángel Casado, Rafael Esteban, María Buti

**Affiliations:** ^1^ Liver Unit, Internal Medicine Department Hospital Universitari Vall d'Hebron Barcelona Spain; ^2^ Department of Medicine Universitat Autònoma de Barcelona Barcelona Spain; ^3^ Pharmacoeconomics & Outcomes Research Iberia (PORIB) Madrid Spain; ^4^ Department of Microbiology Hospital Universitari Vall d'Hebron Barcelona Spain; ^5^ Department of Microbiology Universitat Autònoma de Barcelona Bellaterra Spain; ^6^ CIBERehd Instituto Carlos III Barcelona Spain; ^7^ Biochemistry and Microbiology Department Clinical Laboratories Hospital Universitari Vall d'Hebron Barcelona Spain; ^8^ Liver Pathology Unit, Biochemistry and Microbiology Departments Hospital Universitari Vall d'Hebron Barcelona Spain

**Keywords:** cost‐effectiveness analysis, COVID‐19 pandemic, hepatitis C elimination, linkage to care, lost to follow‐up patients

**Abbreviations**
ALT: Alanine Aminotransferase
AST: Aspartate Aminotransferase
DAA: direct‐acting antivirals
HCV: Hepatitis C virus
LTFU: Lost to follow‐up
RNA: Ribonucleic acid

## 1 INTRODUCTION

Hepatitis C virus (HCV) infection affects 71 million people worldwide. The World Health Organization (WHO) has set a goal of HCV elimination by 2030, and this implies that 90% of HCV cases must be diagnosed and 80% treated.^1^ To improve the diagnosis of HCV, a one‐step approach (HCV‐RNA reflex testing) was introduced in the Spanish National Health System in 2018. A high proportion of patients with diagnosed HCV infection (mainly psychiatric or dependency individuals) are lost to follow‐up (LTFU), likely because of asymptomatic and silent progression of the disease. Retrieval of these patients for treatment could stop further progression of liver disease, reduce the number of infected individuals, and serve as secondary prevention, by averting ongoing transmission.^2^

The ReLink‐C strategy was based on a retrospective search for HCV‐RNA‐positive cases from the central laboratory of Barcelona area between 2019 and 2021 and included the COVID‐19 pandemic. The impact of the COVID‐19 pandemic on global efforts for HCV elimination has been evaluated in several studies,^3^ which show that a one‐year delay in HCV elimination programs is associated with an increase in the number of hepatocellular carcinoma (HCC) cases and liver‐related mortality in the next years.^4^

The aims of this study were as follows: (1) identify and retrieve individuals previously diagnosed with HCV infection who were lost to medical follow‐up, and linked them to care; (2) assess the impact of the COVID‐19 pandemic on the rates of HCV‐positive patient retrieval and linkage to care; and (3) evaluate the cost‐effectiveness of this strategy.

## 2 PATIENTS AND METHODS

ReLink‐C study has a first retrospective phase, focused in identifying HCV‐RNA‐positive cases and a medical records review. The following individuals were excluded as follows: those deceased, those already treated, those with life‐threatening comorbidities or those lacking contact information. The interventional second phase was focused on individuals who had been LTFU. They were called a maximum of five times and sent appointment reminders by post when telephone contact failed. Successfully contacted individuals were informed about our retrieval project and HCV therapy.

In a one‐step visit, a hepatologist recorded the medical history, HCV‐RNA and HCV genotype were performed as well as transient elastography (TE). Severe liver fibrosis was defined by TE between 9.5 and 12.4 kPa and cirrhosis if ≥12.5 kPa. Patients with advanced fibrosis underwent abdominal ultrasound. DAAs therapy was offered to all patients with active infection.

### 2.1 Cost‐effectiveness analysis

An economic evaluation was performed to estimate the cost‐effectiveness of ReLink‐C compared with no intervention. ReLink‐C cost was calculated as the sum of the cost of healthcare resources for the HCV diagnosis and linkage to care.

The impact on healthcare and economic outcomes of ReLink‐C vs no intervention were estimated using a previously reported Markov model^5^ to simulate the evolution of chronic HCV through different health states over the patient's lifetime. The mean age of patients entering the model was 57 years according to our study data, and the distribution between the different health states and sustained virological response (92–97%) was obtained from official data.

### 2.2 Statistical analysis

Relink strategies and linkage to care were compared for the two 14‐month time periods before and after emergency response policies were implemented on 13 March 2020. Descriptive statistics were used to show the differences between the number of patients LTFU who were retrieved and tested in the pre‐COVID and the COVID‐19 period. Statistical analyses were made with EPIDAT 3.1 software. Qualitative data were compared using the chi‐squared test or the Fisher exact test when frequencies were <5.

### 2.3 Ethical considerations

This study was approved by the Research Ethics Committee of Vall d'Hebron Hospital and was conducted following good clinical practice guidelines. All data were processed confidentially in an anonymous database accessible only to the researchers, in keeping with Spanish legislation.

## 3 RESULTS

**FIGURE 1:**
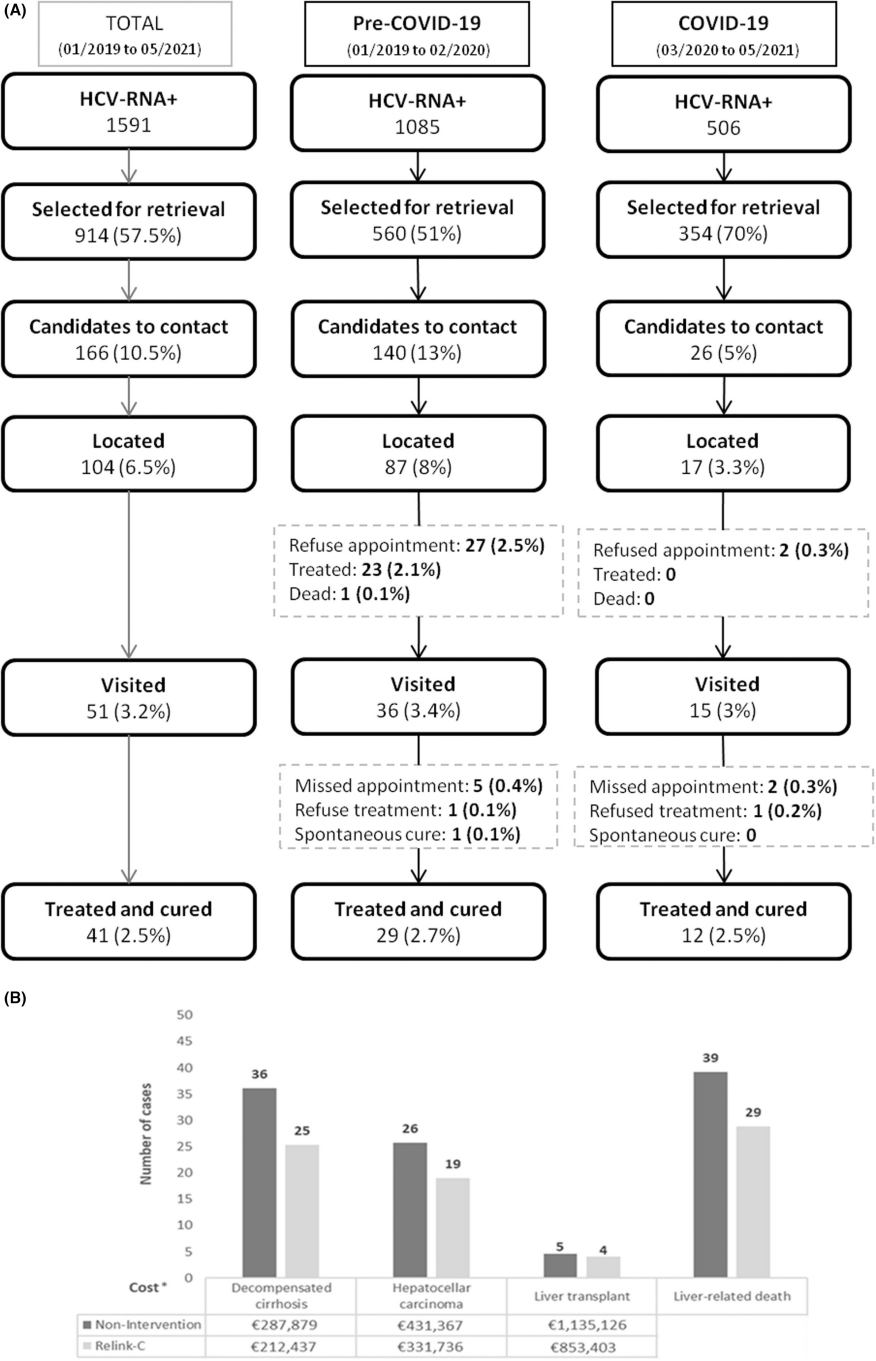
(A) Flowchart of ReLink‐C strategy for the total period and comparison of pre‐COVID and COVID periods. (All percentages are calculated from the total number of HCV‐positive patients identified). Abbreviations: HCV: Hepatitis C virus; RNA: Ribonucleic acid; DAA: Direct acting antivirals. (B) Number of cases of liver complications, mortality and costs projected over the patients' lifetime

In total, 1591 HCV‐RNA‐positive patients were identified. Among them, 599 (37.6%) had already been treated or linked to care, 78 (5%) had died, and 914 (57.4%) had been LTFU and were selected for retrieval. Within the latter, 166 were candidates for contact. Those who were not eligible to be contacted included 379 (41%) with a limited life expectancy, life‐threatening disease, or HCV treatment contraindication, and 369 (40%) lacking contact details. After a maximum of 5 telephone calls, 104 of the 166 candidates (63%) were located, and 51 (50%) agreed to an appointment (29 refused a medical visit, 23 were already treated, and 1 had died). Finally, 43 patients attended the appointment (84%) and 41 were treated (95%) (2.5% of the 1591 HCV‐RNA‐positive individuals identified). A flowchart of the results during the two periods (pre‐COVID‐19 and COVID‐19) is shown in Figure 1A.

During the COVID‐19 period, a lower percentage of patients had been linked to care (27% vs 43%; *p* < 0.0001), and therefore, a higher percentage were selected for retrieval (70% vs 51%; *p* < 0.0001). In contrast, in the pre‐COVID‐19 period, a lower percentage were candidates for contact (5% vs 13%; *p* < 0.0001) due to a limited life expectancy or comorbidities, and a lower percentage were located (3.3% vs 8%; *p* < 0.005). At the end of the analysis, however, a similar percentage of cases started treatment in the two groups (2.4% vs 2.7%; *p* < 0.7239).

Patients treated with DAAs were mainly men (58.5%), mean age 54 years, 10 (25%) had advanced fibrosis and 15 were infected by genotype 1 (37%).

### 3.1 Cost‐effectiveness analysis

The cost of diagnosing all patients who attended the medical visit was €19,924. Therefore, the total investment of the ReLink‐C strategy was €26,075. In the Markov model lifetime simulation, 141 HCV‐RNA‐positive patients were included (166 candidates to contact excluding 23 already treated, 1 death, and 1 spontaneous cure). Based on our results, treating 41 patients with DAAs vs no patients treated (no intervention) reduced decompensated cirrhosis cases by 30%, HCC by 27%, and liver transplantation requirement by 20%. HCV‐related deaths decreased by 26%. The reduction in liver complications generated a cost‐saving of €456,796 (Figure 1B).

## 4 DISCUSSION

The Relink‐C strategy enabled identification of a substantial percentage of HCV patients LTFU and provided linkage to care. With this case‐finding approach, we can prevent potentially severe complications, and reduce further transmission, thereby contributing to HCV elimination. In total, 1591 HCV‐RNA‐positive tests were reviewed, 166 patients were candidates for retrieval, and 25% of these individuals could be relinked to care and cure.

In a similar study in Utrech,^6^ among 1913 individuals with HCV infection, 269 were LTFU and eligible for retrieval. After contacting them by letter, 15.5% of candidates were treated, a lower percentage than in our strategy (25%); suggesting that a phone call may be more effective than a letter for this purpose. In another study,^7^ 499 HCV cases were identified and 3 started DAA (12.5%). In this study, patients were contacted by primary care physicians, a possible reason for the lower rate of linkage to care. Finally, in a study performed in 2 centers in France,^8^ more than 95% of candidates were contacted, due to an active search strategy in which the primary care physicians were involved.

The approach used in our study has several strengths. First, the use of universal reflex HCV‐RNA testing. Second, the simplified care circuit model to assess liver disease. In a single medical visit, HCV‐RNA testing and TE were performed, allowing initiation of DAAs. Of the 43 individuals who attended the appointment, 41 started treatment (>95%).

A novel finding of this study is the observation that during the COVID‐19 period, a lower percentage of patients were linked to care (27 vs 43%), and consequently, more patients were selected for retrieval (70 vs 51%). Nevertheless, the percentage of candidates to contact was lower in the COVID‐19 period, as there was a higher percentage of patients with comorbidities or with a limited life expectancy (33% vs 54%). A higher percentage of patients was located (65% vs 62%) among candidates to contact during the COVID‐19 pandemic, and a larger number of patients located attended the physician's appointment (88% vs 41%) than during pre‐COVID‐19.

Another significant finding to point out is the result of the cost‐effectiveness analysis, which showed a cost‐saving of €456,796 for the public health system because of the estimated reductions in liver disease complications and mortality.

Our study has the limitations of a retrospective design. After the records search, a large percentage of patients could not be contacted due to lacked contact information, a problem observed in other similar studies.^6^, ^7^ In addition, a large geographic area was covered, and the hospitals, primary care centers and drug addiction centers did not have a common record system.

To summarize, individuals with HCV LTFU can be effectively retrieved through the Relink‐C strategy. From the individual's viewpoint, linkage to care enhances HCV cure and from the public health perspective, this strategy contributes to reduce hepatitis C transmission and enables HCV elimination. Although the COVID‐19 pandemic had a relevant impact on linkage to care and treatment, the Relink‐C strategy retrieved and treated a considerable number of formerly lost patients and proved to be a cost‐effective approach in a publically funded universal health system.

## AUTHOR CONTRIBUTIONS

Elena Vargas‐Accarino, Joan Martínez‐Campreciós, Ariadna Rando‐Segura and Maria Buti performed the research. Raquel Domínguez‐Hernández and Miguel Ángel Casado analysed the data. Elena Vargas‐Accarino, Joan Martínez‐Campreciós, Raquel Domínguez‐Hernández and Maria Buti designed the research study and wrote the manuscrit. Mar Riveiro‐Barciela, Francisco Rodríguez‐Frías, Ana Barreira, Adriana Palom and Rafael Esteban contributed to the design of the study. All authors reviewed and approved the final version of the paper.

## CONFLICT OF INTEREST

Raquel Domínguez‐Hernández and Miguel Ángel Casado are employees of Pharmacoeconomics & Outcomes Research Iberia, a consultancy firm specializing in the economic evaluation of healthcare interventions, which has received unconditional funding from Gilead Sciences for the development of the analysis. Dr Mar Riveiro‐Barciela has served as a speaker of Gilead Sciencies and Abbvie. Prof. Rafael Esteban has served as a speaker and advisory board of Gilead Sciencies and Abbvie. Prof. Maria Buti has served as a speaker and advisory board of Gilead Sciencies and Abbvie.

## Data Availability

The data that support the findings of this study are available from the corresponding author upon reasonable request.
